# Dietary Glycaemic Index Labelling: A Global Perspective

**DOI:** 10.3390/nu13093244

**Published:** 2021-09-17

**Authors:** Alan W. Barclay, Livia S. A. Augustin, Furio Brighenti, Elizabeth Delport, C. Jeyakumar Henry, John L. Sievenpiper, Kathy Usic, Yang Yuexin, Andreea Zurbau, Thomas M.S. Wolever, Arne Astrup, Mònica Bulló, Anette Buyken, Antonio Ceriello, Peter R. Ellis, Marie-Ann Vanginkel, Cyril W.C. Kendall, Carlo La Vecchia, Geoffrey Livesey, Andrea Poli, Gabriele Riccardi, Jordi Salas-Salvadó, Antonia Trichopoulou, Kalpana Bhaskaran, David J.A. Jenkins, Walter C. Willett, Jennie C. Brand-Miller

**Affiliations:** 1 Accredited Practising Dietitian, Sydney 2209, Australia; 2 Istituto Nazionale Tumori IRCCS Fondazione G. Pascale, 80131 Napoli, Italy; l.augustin@istitutotumori.na.it; 3 Human Nutrition Unit, Food & Drug Department, Università Degli Studi di Parma, 43121 Parma, Italy; furio.brighenti@unipr.it; 4 Glycemic Index Foundation of South Africa or Glycemic Index Foundation SA, Nelspruit 1201, South Africa; liesbet@gifoundation.com; 5 Singapore Institute of Food and Biotechnology Innovation, Singapore 117599, Singapore; jeya_henry@sifbi.a-star.edu.sg; 6 Department of Nutritional Sciences, Temerty Faculty of Medicine, University of Toronto, Toronto, ON M5S 1A8, Canada; john.sievenpiper@utoronto.ca (J.L.S.); azurbau@inquis.com (A.Z.); cyril.kendall@utoronto.ca (C.W.C.K.); david.jenkins@utoronto.ca (D.J.A.J.); 7 Clinical Nutrition and Risk Factor Modification Centre, St. Michael’s Hospital, Toronto, ON M5B 1W8, Canada; 8 Department of Medicine, Temerty Faculty of Medicine, University of Toronto, Toronto, ON M5S 1A8, Canada; 9 Division of Endocrinology and Metabolism, Department of Medicine, St. Michael’s Hospital, Toronto, ON M5B 1W8, Canada; 10 Li Ka Shing Knowledge Institute, St. Michael’s Hospital, Toronto, ON M5B 1W8, Canada; 11 Glycemic Index Foundation, Glebe 2037, Australia; kathyu@gifoundation.org.au; 12 National Institute of Nutrition for Health, Beijing 100051, China; yuexin_yang@sina.com; 13 INQUIS Clinical Research Ltd., Toronto, ON M5C 2N8, Canada; thomas.wolever@utoronto.ca; 14 Healthy Weight Center, Novo Nordisk Foundation, Tuborg Havnevej 19, DK 2900 Hellerup, Denmark; ARA@novo.dk; 15 Departament de Bioquímica i Biotecnologia, Universitat Rovira i Virgili, 43003 Reus, Spain; monica.bullo@urv.cat (M.B.); jordi.salas@urv.cat (J.S.-S.); 16 Institut d’Investigació Pere Virgili (IISPV), Hospital Universitari de Sant Joan de Reus, 43204 Reus, Spain; 17 Centro de Investigación Biomédica en Red Fisiopatología de la Obesidad y la Nutrición (CIBEROBN), Instituto de Salud Carlos III (ISCIII), 28029 Madrid, Spain; 18 Institute of Nutrition, Consumption and Health, Paderborn University, 33098 Paderborn, Germany; anette.buyken@uni-paderborn.de; 19 IRCCS MultiMedica, 20099 Milan, Italy; antonio.ceriello@hotmail.it; 20 Biopolymers Group, Departments of Biochemistry and Nutritional Sciences, Faculty of Life Sciences & Medicine, King’s College London, London SE1 9NH, UK; peter.r.ellis@kcl.ac.uk; 21 School of Sport and Health Sciences, University of Brighton, Brighton BN2 4AT, UK; M.Vanginkel@brighton.ac.uk; 22 Department of Clinical Sciences and Community Health, Università Degli Studi di Milano, 20122 Milano, Italy; carlo.lavecchia@unimi.it; 23 Independent Nutrition Logic Ltd., Wymondham NR18 0QX, UK; glivesey@inlogic.co.uk; 24 NFI—Nutrition Foundation of Italy, 20124 Milan, Italy; poli@nutrition-foundation.it; 25 Department of Clinical Medicine and Surgery, Federico II University, 80147 Naples, Italy; riccardi@unina.it; 26 Hellenic Health Foundation, 115 27 Athens, Greece; atrichopoulou@hhf-greece.gr; 27 Glycemic Index Research Unit, Centre for Applied Nutrition Services, Temasek Polytechnic, Singapore 52975, Singapore; Kalpana_BHASKARAN@TP.EDU.SG; 28 Departments of Nutrition and Epidemiology, Harvard T. H. Chan School of Public Health and Harvard Medical School, Harvard University, Boston, MA 02115, USA; wwillett@hsph.harvard.edu; 29 School of Life and Environmental Sciences and Charles Perkins Centre, University of Sydney, Camperdown, Sydney 2006, Australia; jennie.brandmiller@sydney.edu.au

**Keywords:** glycaemic index, diabetes, food labels, food regulation

## Abstract

The glycaemic index (GI) is a food metric that ranks the acute impact of available (digestible) carbohydrates on blood glucose. At present, few countries regulate the inclusion of GI on food labels even though the information may assist consumers to manage blood glucose levels. Australia and New Zealand regulate GI claims as nutrition content claims and also recognize the GI Foundation’s certified Low GI trademark as an endorsement. The GI Foundation of South Africa endorses foods with low, medium and high GI symbols. In Asia, Singapore’s Healthier Choice Symbol has specific provisions for low GI claims. Low GI claims are also permitted on food labels in India. In China, there are no national regulations specific to GI; however, voluntary claims are permitted. In the USA, GI claims are not specifically regulated but are permitted, as they are deemed to fall under general food-labelling provisions. In Canada and the European Union, GI claims are not legal under current food law. Inconsistences in food regulation around the world undermine consumer and health professional confidence and call for harmonization. Global provisions for GI claims/endorsements in food standard codes would be in the best interests of people with diabetes and those at risk.

## 1. Introduction

In line with the recommendations of the United Nations Food and Agriculture Organisation [1], Nutrition Information and/or Facts Panels are provided on the labels of packaged foods in many developed and developing nations to help consumers make informed choices. Although carbohydrates are a mandatory component [2], the only requirement in many countries is the total carbohydrates per 100 g or per 100 mL and/or serve of food, and this may or may not include dietary fibre, depending on the jurisdiction. In some cases, the total sugars, added sugars and/or dietary fibre are also included. In most nations, ingredient lists are also mandated on the labels of packaged foods, and these incorporate the names of common carbohydrate-containing ingredients. Consumers are able to utilize this information to make informed food purchasing decisions at the point of sale.

However, this limited information does not assist people with diabetes or pre-diabetes and others who are required to manage blood glucose levels. One consequence is that many consumers choose to restrict foods containing carbohydrates [3], particularly those containing sugars [4,5] and including those containing dietary fibres, in certain jurisdictions. Globally, 1 in 11 adults has diabetes, or approximately 463 million people [6].

The glycaemic index (GI) is an inherent property of carbohydrate-containing foods and beverages [7]. On a scale from 1 to 100 (glucose scale), this index reflects the food matrix within which the available (glycaemic) carbohydrate is embedded and the rate at which this carbohydrate is digested, absorbed and metabolised and, thus, appears as glucose in the blood. Low GI foods (≤55) are characterised by a smaller incremental rise and fall in blood glucose. High GI foods (≥70) are characterised by faster and higher peaks and troughs in blood glucose levels [8].

The GI of a food is not predicted on the basis of nutrient composition or ingredient list but is derived from laboratory testing in human subjects. Multiple factors contribute, including the relative proportions of the available carbohydrate (i.e., sugars or starches), the types of sugars (e.g., fructose, glucose, maltose and sucrose), the botanical source of the starch and its physico-chemical characteristics (e.g., the degree of gelatinization and amylose and amylopectin content), the particle size of the food, lipid- and protein-starch interactions, the presence of viscous fibres, encapsulation by intact cell walls (“fibre matrix”) and other factors that make up the composition and structure of individual foods and how they are processed [9,10,11]. 

Similarly to the GI, dietary fibre represents a diverse and heterogeneous group of mostly carbohydrate polymers that vary in their functional properties and are often defined by their physiological effects (i.e., laxation, reduced cholesterol or blood glucose) [2]. However, dietary fibre is a mandatory component of Nutrition Facts panels in North America and is voluntarily included in other regions.

The glycaemic load (GL) is defined as the product of the amount of available (glycaemic) carbohydrate per serve multiplied by the GI of a food or beverage (GL = Carbohydrate(g) × GI/100), which, when describing foods has units of grams (g) of GL per serving [12]. Brand-Miller and colleagues suggested that high GL foods and beverages are defined by having a GL value of 20 g or more per serving; medium GL foods and beverages have a GL value between 11 and 19 g per serving; and low GL foods and beverages have a GL value of 10 g or less per serving [13].

For epidemiological studies, the GL is adjusted for the energy intake, in line with other macronutrients and fibre [14]. However, for food labelling purposes, GI is typically preferred over GL, because serving sizes vary from product to product in most countries and because macronutrients are not adjusted for energy.

A large body of evidence has accumulated over the last four decades showing that low GI or GL diets assist with diabetes prevention [15,16] and management [17]. Many diabetes associations now recommend the consumption of healthy carbohydrate-containing foods with a low GI [18,19,20]. However, unlike the total carbohydrates, few nations specifically regulate the inclusion of GI or GL information in food and drink Nutrition Information or Facts panels, or in associated nutrition, health or related claims.

To date, nutrition, health and related claims [2] have been voluntarily included on food labels in a number of different ways:

(a) Nutrition content claims. These are claims that are about the presence or absence of, a macronutrient (protein, fat, carbohydrate or fibre), energy, a micronutrient (vitamin or mineral), or biologically active substance.
(b) Health claims. These are claims that state, suggest or imply that a food or a property of the food has, or may have, a health effect (biochemical, physiological or functional process or outcome).
(c) Endorsements. These are nutrition content claims or health claims that are made with the permission of an endorsing body, which is a not-for-profit entity that has a nutrition- or health-related purpose or function.

In addition to these traditional claims, front-of-pack labelling schemes are being developed and voluntarily introduced in many nations around the globe. While they are designed to help people make quick food purchasing decisions at the point of sale, they are based on information from the Nutrition Information or Facts panels. Therefore, the majority of people with diabetes and those at risk do not have access to information about the food or beverages effect on glycaemia at the point of sale.

The aim of this review is to summarise the current GI and GL labelling practices around the globe, identifying the type of claims being made in major jurisdictions and where claims are not specifically permitted within food regulations explaining the rationale why.

## 2. Materials and Methods

We undertook a narrative review of the evidence of GI and GL labelling practices around the world. We searched the scientific literature (e.g., PubMed), food and beverage regulatory authority websites and other online resources (e.g., Food and Agriculture Organisation of the United Nations) and contacted other sources of information (e.g., government and professional contacts within major regions) that were relevant to this aim. Our review included any information on food labels related to the GI, including GI nutrient content claims, health claims, endorsements, logos or other front-of-pack labelling tools. All countries with information about GI on food labels were included.

## 3. Results

### 3.1. Australia and New Zealand

Low GI nutrition content claims began appearing on Australian food and beverage labels in the late 1990s, after the publication of ‘The G.I. Factor’ book [21] in 1996. GI nutrition content claims were permitted on Australian food and beverage labels provided, as for all such claims, that they were not false or misleading. At this point in time, however, GI claims were not specifically regulated under Australia’s Food Standards Code or Code of Practice on Nutrient Claims.

A new standard for nutrition, health and related claims was officially gazetted in Food Standards Australia New Zealand’s (FSANZ) Food Standards Code in 2013 [2]. It defined GI as “…*a measure of the blood glucose raising ability of the digestible carbohydrates in a given food as determined by a recognised scientific method*”, and this is regulated as a nutrition content claim with special conditions (Table 1).

In Australia and New Zealand, the Nutrient Profile Scoring Criterion (NPSC) [2], is a complex algorithm that takes into account the food group (three broad categories of beverages; fats, oils and spreads; and all other foods), energy density, saturated fat, total sugars, sodium, protein, fibre, fruits, vegetables, nuts and legume content per 100 g or 100 mL of the food or beverage, respectively. This profiling is intended to ensure that higher level nutrition claims are only able to be made on healthier foods and beverages.

In addition to GI nutrition content claims, the GI Symbol is a Certification Trademark (CTM) that represents a food endorsement scheme with strict nutrient criteria, including the requirement to show a GI value in the Nutrition Information panel. It has featured on the labels of foods and beverages since 2002. Initially, the GI Symbol was registered by the University of Sydney and licensed to the GI Foundation, a not-for-profit entity with three partners: the University of Sydney, Diabetes Australia and the Juvenile Diabetes Research Foundation Australia. 

**Figure 1:**
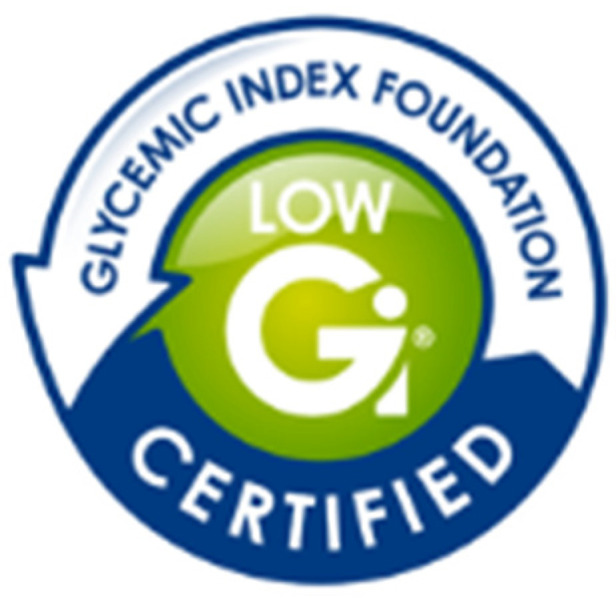
Australia and New Zealand’s Glycaemic Index (GI) Symbol.

The current version of the GI Symbol (Figure 1) is now registered and owned by the GI Foundation in countries outside Australia and New Zealand, including North America, South America, the EU and select parts of Asia. Under Australia and New Zealand’s Food Standards Code it is permitted as an endorsement because its owner, the Glycemic Index Foundation, is an “Endorsing body” [2].

In order to utilise the GI Symbol, foods must have a low GI determined by a standardised methodology (ISO 26642:2010) [8] and also meet stringent nutrient criteria for the energy (kilojoules), total available (glycaemic) carbohydrates, saturated fat, sodium and, in certain foods, fibre and calcium [22]. These nutrient criteria are in line with FSANZ’s Nutrient Profile Scoring Criteria, as well as international dietary guidelines [23]. Food nutrient profiling has demonstrated that the GI Symbol Program’s Product Eligibility and Nutrient Criteria correlate very strongly with FSANZ NPSC and Australia’s Health, Star Rating system [24].

In Australia, there is evidence of practical implementation. When the GI Symbol was launched in 2002, five foods/beverages utilized the GI Symbol on packaging. This increased to over 300 foods/beverages (stock-keeping units) by 2021. Prior to the launch of the GI Symbol, market research was conducted in Australia by Newspoll (Newspoll Market Research 2002) and then annually until 2012 (ACNielsen 2012). Participants were main grocery buyers representative of the Australian population (aged 18+ years) and living in the five mainland state capital cities of Australia (Adelaide, Brisbane, Melbourne, Perth and Sydney).

In 2002, 28% of 490 respondents were aware of the GI concept. This increased to 86% of 458 respondents by 2005 and has remained approximately the same since then. Awareness of the GI Symbol was 2% at baseline (Newspoll Market Research 2002) and increased to 37% by 2012 (*n* = 1502) (ACNielsen 2012). Most (94%) consumers who were aware of the GI looked for the GI Symbol when shopping (ACNielsen 2012). The majority (80%) believe that the GI Symbol indicates that foods that carry it are “healthy, wholesome and a good choice”, “scientifically tested” and “provide sustained energy/glucose release”.

A recent survey of 1235 Australians aged 18–75 years suggested that half of consumers considered the presence of the GI symbol as helpful for making food purchasing decisions (Lonergan Research 2016). Of the participants surveyed, 84% indicated that they have an interest in finding out more about how the GI of foods can improve their overall health. Highest awareness of the GI Symbol was amongst people living with type 2 Diabetes (*n* = 273). Once consumers learn more about low GI claims and the GI Symbol, over 65% are likely to look out for it when shopping. They are more likely to look for foods/beverages with the Symbol when they know GI Symbol Certified products satisfy nutritional criteria and have been tested at an accredited laboratory.

There is evidence that the population’s average dietary GI and GL has decreased since the introduction of GI claims on food labels in Australia in the late 1990s. Yeung and colleagues [25] investigated changes in dietary GI and GL in Australian children aged between 2 and 16 years for the years 1995 to 2011–2012. Dietary GI and GL decreased significantly by 2.2% (Ptrend < 0.001) and 11.4% (Ptrend < 0.001), respectively, over the 16.5 year timeframe. The decrease was primarily due to decreasing trends (Ptrend < 0.001) in the mean percentage GL contribution of breads and bread rolls, fruit and vegetable juices, breakfast cereals (ready to eat), potatoes, sweetened beverages, sugar, honey and syrups, frozen milk products and milk and milk product-based dishes. Conversely, there were increasing trends (Ptrend < 0.001) for flours, cereal grains and starches, savoury biscuits, fancy breads, pome fruits, tropical and subtropical fruits, poultry-based dishes and cereal-, fruit-, nut- and seed-bar groups. 

Similarly, Kusnadi and colleagues [26] investigated changes in dietary GI and GL in adults aged 18+ years for the years 1995 to 2011–2012. Dietary GI and GL decreased significantly by 4.6% (Ptrend < 0.001) and 11.7% (Ptrend < 0.001), respectively, over the 16.5 year timeframe. The decrease was primarily due to decreasing intakes (*p* < 0.001) of bread and bread rolls, potatoes, sugar, honey and syrups and pastas as well as increases in cereal-based dishes, flours, cereal grains and starches, pome fruits, savoury biscuits, chocolates and breakfast cereals (hot porridge).

Type 2 diabetes prevalence rates continue to increase in Australia, like most places in the world. However, prevalence is affected by both risk of developing diabetes and survival of those with existing diabetes, and therefore diabetes incidence is a better metric to understand the trends in population risk of diabetes. A recent population-based analysis indicates that type 2 diabetes incidence rates in Australia have been declining relatively quickly since 2010 [27], and this may be at least in part due to improvements in carbohydrate-containing food choices and the associated decrease in population dietary GI and GL.

### 3.2. Africa

South Africa is the only African nation that currently has provisions for GI claims on food labels. In 2002, The Department of Health’s Regulation 1055 REGULATIONS RELATING TO THE LABELLING AND ADVERTISING OF FOODSTUFFS [28] defined GI as “*the blood glucose responses of carbohydrate foods and is defined as the incremental area under the blood glucose response curve of a 50 g carbohydrate portion of a test food expressed as a percentage of the response to the same amount of carbohydrate from pure glucose taken by the same subject*”.

The legislation also specifies that GI nutrition content claims, if used:

i. may only be used for foodstuffs with a total glycaemic carbohydrate content of 40% or more of the total energy value of the foodstuff; and
ii. may, if used, only be indicated as low, intermediate or high glycaemic index or low, intermediate or high GI, in the table with nutritional information or when used as part of a logo, provided the Glycaemic Index category corresponds with certain conditions (Table 2.)

In 2011, regulation 1055 was revoked, and it was replaced by the Department of Health’s Regulation 146 REGULATIONS RELATING TO THE LABELLING AND ADVERTISING OF FOODSTUFFS [29]. It neither explicitly prohibits nor explicitly permits GI claims on food labels. Since then, GI claims have been included in the draft Regulation 429 labelling regulations, published in May 2014, which are due for gazettal soon.

**Figure 2:**
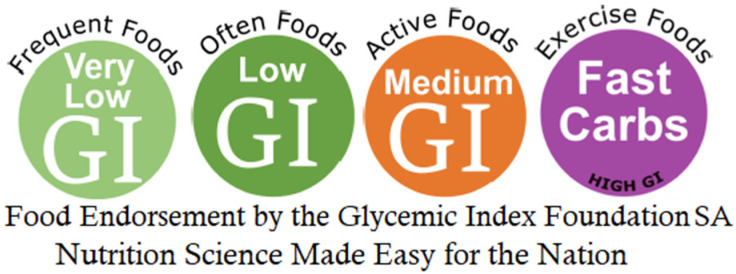
Glycaemic Index Foundation of South Africa’s GI Symbols.

Independent of this, South Africa’s Director General of Health gave the Glycaemic Index Foundation of South Africa (GIFSA) authorisation to act as an independent endorsement entity in 2011. GIFSA was founded in 1999 and, as such, was the first independent organisation in the world to specifically promote GI related labelling on food packaging. GIFSA is a for-profit organisation wholly owned by Jan and Elizabeth Delport. In October 2000, the first food was endorsed with the GIFSA Often Foods endorsement logo (Figure 2), and, in 2020, there were 180 endorsed products.

Four variations of the GIFSA logo distinguish between “Frequent Foods” (very low GI, low fat), “Often Foods” (low GI, lower fat), “Active Foods” (intermediate GI, medium fat) and “Exercise Foods” (high GI). Since 2000, all products that use the GIFSA endorsement have to comply with a broader set of specifications, i.e., total fat, saturated fat, mono-unsaturated fats, cholesterol, sodium, protein, fructose and sugar alcohols, GI (determined by standardised methodology [ISO 26642:2010]) and GL per serving, (GI Foundation SA, 2020).

### 3.3. Asia

#### 3.3.1. China

GI research began in China in the 1980s. In the late 1990s, the National Institute for Nutrition and Health organized a large-scale study on the GI of common foods and published the results in the book “China Food Composition Table”. In 2008, GI was used on food labels in one city’s food guide (DB31/T 399-2008) for packaged cereal foods. In 2011, the national nutrition label was released, invalidating the city’s food guide.

With the increase in diabetes prevalence rates, consumer awareness of the GI and the demand for healthy food choices also increased, and in 2019, the National Health Commission of China issued the first national food standard for measuring the GI of foods, entitled “Standard for Determination of Food Glycaemic Index (GI), WS/T 652—2019”. 

It defines the GI as a “*Property of the carbohydrate in different foods, specifically refers to the two-hour incremental area under the blood glucose response curve (iAUC) after consumption of the carbohydrate portion of a test food (usually 50g) expressed as a percentage of two-hours iAUC after consumption of the same amount of carbohydrate from a reference food (glucose solution)*”. The standard was developed by the National Institute for Nutrition and Health of China CDC.

Only products containing at least 7.5 g of carbohydrate per serving or where carbohydrate accounts for 80% of the macronutrients, are eligible for GI testing. The GI test needs to be conducted according to the WS/T 652—2019 by an accredited laboratory. In addition to meeting these criteria, GI testing of the end product is required, and the GI value of the end product must be <55 to be defined low GI.

In order to promote the development of healthy products by food industry, in 2020, the Chinese Nutrition Society issued the “Standard for GI claims of pre-packaged food T/CNSS 012-2019” for product Certification. To qualify, food products need to fulfil specific nutrient criteria for each food category, that include limiting energy and nutrients like total fat, saturated fat, total sugars, sodium, etc. The “Low GI” message is currently possible for several different food categories, which are further divided into sub-categories.

#### 3.3.2. Singapore

**Figure 3:**
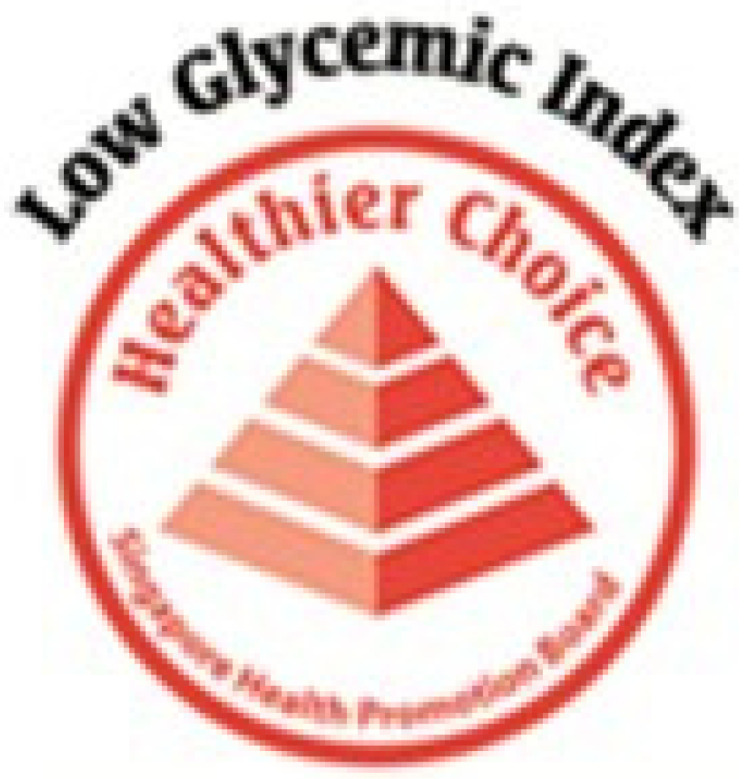
Singapore’s Healthier Choice symbol with the low Glycaemic Index claim.

In 2001, the Singapore Health Promotion Board was established to coordinate the government’s commitment to promote healthy living. Soon after, the Healthier Choice Symbol (Figure 3) was introduced, and it now applies to 3500 packaged foods [30]. There are category-specific nutrient criteria so that food products that are allowed to carry the symbol are lower in fat, saturated fat and trans-fat, sodium and sugars and, where appropriate, are good sources of calcium or wholegrains. To date, there are guidelines covering 18 food categories.

In 2009, Temasek Polytechnic set up the first Glycemic Index Research Unit (GIRU) in Singapore, and a working group involving members from the Health Promotion Board, Temasek Polytechnic and the Singapore Accreditation Council was formed to establish functional food testing guidelines that included GI testing. In 2014, the low version of the GI Healthier Choice Symbol (HCS) was released and the Health Promotion Board strongly encouraged the label to be used on front of pack labels. Initially, the low GI logo was only allowed to be placed on wholegrain cereal products that satisfied the HCS criteria. In 2016, the guidelines were reviewed, and after concerted efforts from food industry and key representatives, the GI logo was extended to all categories of foods in April 2020.

Foods claiming to have a low GI must first qualify for the HCS category-specific nutrient guidelines before applying for the low GI claim. In addition, the foods carrying the HCS Low GI claim must fulfil the following criteria: (1) food products must have a GI value < 55; and (2) food products must contain at least 7.5 g of carbohydrate per serving of the food product or 50% of the macronutrient must be carbohydrate. Products that do not meet the carbohydrate criteria for GI testing are evaluated on a case by case basis. In Singapore, at the national level, new health claims are subject to an application process. Applications can be made for new nutrition function claims with the applicant required to provide information for substantiation. 

**Figure 4:**
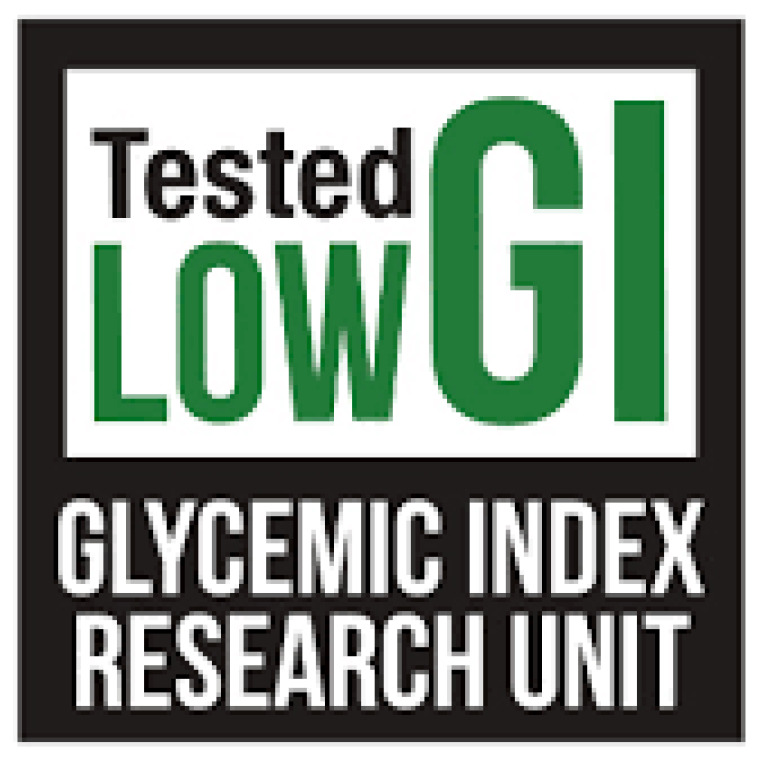
Temasek Polytechnic’s low GI tested logo.

In addition, all the products tested in Singapore by the Glycemic Index Research Unit at Temasek Polytechnic carry the low GI tested logo offered by the Singapore Accreditation Council’s accredited facility (Figure 4). In addition, Enterprise Singapore (previously named as SPRING), offered grants to Small Medium Enterprises to conduct GI testing, which has helped create awareness among the public and has been instrumental in inspiring local manufacturers to develop new low GI products.

In other South East Asian countries, such as Vietnam, Thailand and the Philippines, food products must undergo product registration before they can be marketed. During this process, a low GI nutrition content claim on the label would be evaluated and approved on a case-by-case basis by the Food Authorities in those countries. In other parts of South East Asia, low GI claims vary from country to country (Table 3).

### 3.4. North America

#### 3.4.1. Canada

Low GI diets have been recommended for the prevention/management of diabetes in clinical practice guidelines from Diabetes Canada (formerly, the Canadian Diabetes Association) since the 1990s [18,31,32,33,34]. More recently, low GI diets have also been recommended by clinical practice guidelines for the prevention and management of cardiovascular disease from the Canadian Cardiovascular Society [35] and the Canadian Cardiovascular Harmonized National Guidelines Endeavour [36] and obesity and its complications from Obesity Canada [37].

Despite the endorsements by the major obesity, diabetes and heart associations in Canada, Health Canada published a position against the use of GI claims on food labels in 2011 [38]. The main arguments against GI Labelling were (1) insufficient accuracy and precision of the GI measurement for purposes of labelling; (2) lack of consideration of the quantity of carbohydrate or food consumed and the partial replacement of available with unavailable carbohydrate; and (3) incongruence with national nutritional policies and guidelines. 

These criticisms have been addressed [39,40,41,42] as follows: 

(1) the GI measurement has sufficient accuracy and precision to differentiate between low and high GI foods and would satisfy Canadian Food Inspection Agency labelling tolerances for carbohydrate and fibre of ±20% [43] for the purposes of labelling a food as “low GI” [39,40,41,42]. 

(2) Health Canada was correct in asserting that the glycaemic response depends both on GI and the amount of carbohydrate consumed, but failed to recognize that the relationship between the glycaemic response (GR–In this context, GR is the incremental area below the glycaemic response curve over 2 h) and the amount of available carbohydrate (avCHO) consumed is not linear, but falls off as carbohydrate intake increases [42]. 

Health Canada argued that a low GI food could have a higher GR depending on how much is consumed and attempted to prove this by comparing a serving of spaghetti (GI = 49, 48 g avCHO, GL = 23.5 (The values for GI and avCHO shown here are those ascribed to spaghetti by Health Canada)) to a serving of mashed potatoes (GI = 82, 25 g avCHO, GL = 20.5 (The values for GI and avCHO shown here are those ascribed to potatoes by Health Canada)). It was asserted that, since the GL of spaghetti was higher, it would elicit a higher glycaemic response [38]. However, a 50 g avCHO serving of spaghetti actually elicits a significantly lower GR than a 25 g avCHO serving of mashed potato [44]. The explanation for this and further illustration of the lack of proportionality between GL and GR, can be found elsewhere. 

(3) Unfortunately, many foods bearing nutrition or health claims (e.g., high-fibre cookies or whole grain chips) run counter to national nutritional policies and guidelines. Nutrient profiling criteria used by Health Canada for the existing health claims framework can be applied to any food that might carry a low-GI claim. For example, the criteria used to support the oat products and cholesterol reduction claim require foods be limited in sodium, saturated or trans-fat and alcohol and contain at least 10% of the recommended weight of a vitamin or mineral [39,40,41,45].

In place of GI labelling, in 2013 Health Canada began to develop a framework for claims related to the relative reduction in postprandial glycaemic response as a means to address the perceived limitations of GI labelling [46]. The framework proposed to allow health claims without premarket approval for a relative reduction in the postprandial glycaemic response (similar to the relative response used in the calculation of GI) that, unlike GI, was not based on the response to a single universal reference food such as glucose or white bread but rather a specific reference food that did not have the added or substituted ingredient or multiple-compositional changes or inherent properties used to effect the reduction in postprandial glycaemic response [46]. 

The consultation period closed in September 2013 with no subsequent follow up response or action from Health Canada [46]. Some advancement, however, occurred out of consultations between Health Canada and Pulse Canada in 2016, resulting in a permissible claim for pulses: “One cup (250 mL) of cooked (type of whole pulse) in place of [instead of] low fibre starchy foods results in a reduced blood sugar [glucose] rise after a meal.” [47].

An interest in GI labelling per se persisted despite the framework being developed for claims related to the relative reduction in the postprandial glycaemic response. A “third way” for GI labelling arose out of discussions between Health Canada, University of Toronto researchers and Diabetes Canada in 2014. The approach involved the development of a symbol program that would operate outside the current health claims framework. 

Diabetes Canada was the logical choice to host the symbol program as the program would represent the ultimate knowledge translation of its Clinical Practice Guidelines, which had a long history of recommendations of low-GI diets for the prevention and management of diabetes [18,31,32,33,34]. In June 2015, a press release was issued expressing Diabetes Canada’s intent to pursue a low-GI education and symbol program with the support of University of Toronto researchers and Health Canada acting in a permissive and advisory role [48].

Since then, Diabetes Canada has commissioned consumer research and undertaken a broad set of consultations to help define the focus of a program based on low-GI versus carbohydrate quality. The research showed that Canadians, independent of their health history, were interested in a consumer-facing symbol program that focused on low-GI carbohydrate foods that would complement other measures of carbohydrate quality, such as dietary fibre and the promotion of whole foods [49]. 

The consultations included a “Canadian Diabetes Association Session on Glycaemic Response/Index/Load in Diabetes” at the 33rd International Symposium on Diabetes and Nutrition in Toronto, Canada on 11 June 2015 that coincided with the press release announcing Diabetes Canada’s intent to pursue the low-GI education and symbol program [50,51] and a joint “Workshop on The Scientific Basis for Communicating Carbohydrate Quality” hosted by ILSI North America and Diabetes Canada in Washington DC on 2 February 2017 [52]. 

These sessions brought together Diabetes Canada with academic researchers, Health Canada, the European Food Safety Authority, the Food and Drug Administration (FDA), the American Diabetes Association, the Glycemic Index Foundation, the Whole Grain Council (which administers the Whole Grain Stamp [53]), industry and trade groups. Together, the research and consultations reinforced the decision to pursue the low-GI education and symbol program.

As an early step in the development of the program, Diabetes Canada launched a Glycaemic Index Education Portal [54], The Glycaemic Index Educator’s handbook [55], Glycaemic Index Food Guide with a Stop Light System [56] and Glycaemic Index Food Cards [57] in 2017. Since then, there has been no further progress.

#### 3.4.2. USA

The Food and Drug Administration (FDA) is responsible for regulating the labelling of foods sold in the United States of America as governed under the Food, Drug and Cosmetic Act (FD&C Act) and the Fair Packaging and Labelling Act Federal laws. The FDA has defined three categories of claims: nutrient content claims, structure/function claims and health claims. Nutrient content claims characterise the nutrient profile of the food within defined regulatory requirements. For example, “sugar free” is a permitted nutrient claim for food with <0.5 g of sugar per Reference Amount Customarily Consumed. 

Structure and function claims cannot imply relation to a disease but rather to the effect a nutrient or ingredient has on maintaining or supporting physiological processes. This broad categorization was intended for application in dietary supplements while conventional foods were limited to effects derived from the nutritive value of the food. However, the FDA has acknowledged the limitation in defining “nutritive value” and that a conceptual framework, which distinguishes structure function claims of conventional foods from dietary supplements is needed. 

Until “nutritive value” is defined, structure/function claims for conventional foods follow the guidance for dietary supplements. However, manufactures are not obliged to notify the FDA of a claim nor include disclaimers that the claim has not been evaluated by the FDA and that the product is not intended to prevent, mitigate, diagnose cure or treat any disease. Health claims mark the relationship between a food or nutrient to a disease and can either be supported by “Significant Scientific Agreement” or be allowable by the FDA as a qualified health claim on a case-by-case basis upon petition to the FDA.

Despite the nutrition recommendations of the American Diabetes Association [58], to date, glycaemic index is not defined by the FDA for regulatory labelling. Statements related to GI are not considered to be nutrient content, as in their opinion, they do not describe the content of a nutrient in the food, structure/function, nor health claims. As a result, GI claims may be permitted under the general false and misleading provisions of the FD&C Act, which mandates that all labelling is truthful, evidence-based and not misleading. 

Several GI Certification programs, such as the GI Symbol Program of the Glycemic Index Foundation (Australia), are also available in the USA. While the FDA has not pre-approved participation in these certification programs, no action against manufactures who utilise low GI logos, either as part of a program or developed by the manufacturer, has been noted. Currently, GI testing protocols may be acceptable for claims related to postprandial glycaemia. These claims are categorised as structure/function claims, must be evidence-based and cannot imply a relation to a disease, inclusive of utilising terminology characteristic of a disease, such as “reduces blood sugar levels”, which can be perceived as diabetes management.

#### 3.4.3. Europe

In Europe, the use of GI in food selection is infrequently endorsed by the bodies issuing dietary recommendations. In 2010, the European Food Safety Authority (EFSA), which is an independent body acting as the scientific support to risk managers of the EU government and member states, issued the European Dietary Reference Values (DRV) document for carbohydrates [59]. The panel found the evidence for GI inconclusive and, therefore, made no specific recommendations. However, agencies and advisory bodies of some European countries have taken the GI into consideration when preparing national DRV documents on carbohydrates, albeit with contrasting results (Table 4).

In addition to the limited support for the GI concept in national reports, daily reference values (DRVs) and food-based dietary guidelines, proposing a low GI label within the set of regulations currently existing in the EU presents further challenges. Labelling a food with its GI properties might be considered a health claim for the general population. According to the EU regulation 1924/2007, the criteria for a health claim on the effect of foods or food ingredients are straightforward: the effect must be a beneficial physiological one and the active ingredient to which the effect is related should be clearly identified and characterised. 

Regarding the beneficial physiological effect, specific guidance was issued by EFSA in 2012 about the requirements for health claims related to blood glucose concentrations [65]. A lower postprandial glycaemic response was considered a beneficial physiological effect, provided that insulin was not disproportionally increased. Regarding the need for food characterisation, the component that results in a health effect (e.g., lower glycaemia) should be indicated. Since then, a number of well-characterised food ingredients have been authorised to bear the postprandial glycaemic response reduction claim, including sugar alcohols, fructose, resistant starch and some types of dietary fibres, as well as the presence of Slowly Digestible Starch (SDS) (EU registry of authorised health claims [66]). However, a low GI food cannot be always characterised in this manner (e.g., pasta and bread contain the same ingredients despite different GI values).

In November 2018, the European Parliament submitted a question (E-006064) to the EU Commission, asking whether the Glycaemic Index food labelling falls under the Health Claims Regulation 1924/2007 and, if not, to indicate the provisions that apply to GI labelling.

In January 2019 the commission answered that “*claims referring to the Glycaemic Index of a food are falling under the scope of Regulation (EC) No 1924/2007” […]*. *Carbohydrates* [foods] *with a low GI are not sufficiently characterised* [but] *in the past EFSA assessed several health claims on the reduction of postprandial glycaemic response […], with a favourable outcome reflected by the authorised wording of the corresponding permitted health claims.* [Therefore] *Member States may allow GI-claims accompanied by or similar to the authorised claims on postprandial glycaemic response* *within the limits set by Regulation 1924/2007*.”

This is the same approach followed by the Italian Ministry of Health, which, in 2017 issued a interpretative letter indicating that, if a food has received formal approval for a health claim on the reduction of the postprandial glycaemic response (and only on this condition), it may be possible to use the GI information on the food packaging (i.e., Nutrition Information panel), although GI nutrition content claims per se are not allowed.

In our view, the ability to select carbohydrate foods that are naturally low-GI remains an important issue for consumers with diabetes and pre-diabetes. The Food Information for Consumers (FIC) 1169/2011 EU Regulation, encourages information to consumers to be included on food labels. This opens up the possibility for voluntary forms of expressions, such as the Keyhole interpretive logo endorsed by Scandinavian countries. Article 36 of the FIC Regulation lists requirements of voluntary food information, including (3-c) *the indication of reference intakes for specific population groups in addition to the reference intakes set out in Article XIII*, provided that information is not misleading, not ambiguous for the consumer and based on relevant scientific data [66].

Our review also found that associations of individuals with diabetes, as well as scientific societies, are paramount in supporting the need for voluntary labelling of GI values. This information was presented by the International Carbohydrate Quality Consortium (ICQC) to the EU Parliament on the 5th of February 2019. The request for resolution motion from the European Parliament to the European Commission, was under article 133 of the code and is transcribed as follows:

“***A.*** *considering that several scientific studies show that diets which avoid high glycaemic peaks after meals are associated with lower risk of type 2 diabetes, CVD and obesity;*

***B.*** *considering that one of the simplest and most efficient methods to control postprandial glycemia is slowing the dietary carbohydrate absorption by choosing low glycaemic index (GI) foods;*

***C.*** *considering that, in order for consumers to make informed choices, some extra-European countries allow labelling foods that have low GIs and healthy nutritional profiles in line with dietary guidelines, and that such labelling appears to improve the well-being of the general population and in particular of people with diabetes;*

***D.****asks the European Commission to adopt the necessary measures to guarantee the presence of a low glycaemic index symbol (Low GI) on the label of food products in Europe in order to improve healthy food choices with the aim of reducing the risk of diet-related disease and complications, and health costs in Europe*.”

### 3.5. Global Summary

A summary of the global GI food labelling situation is presented in Table 5.

## 4. Discussion

The global regulatory approach to glycaemic index (GI) claims on food labels can best be described as inconsistent. GI nutrition content claims and/or endorsements are specifically permitted or regulated in some countries, including Australia, India, New Zealand, Singapore and South Africa. General provisions allow them to be utilized in other regions, including China and the USA. They are not permitted in Canada and the European Union. This inconsistency creates confusion and does not serve the best interests of people with diabetes, or those at risk who represent up to one in three adults in certain nations [6]. It also creates barriers to global free trade for food manufacturers/distributors who decide to utilize low GI claims on product labels where permitted.

The need for GI labelling should be evaluated in the light of updated scientific evidence. Systematic literature reviews and associated meta-analyses of randomised controlled trials indicate that substitution of high GI foods/meals with low GI equivalents leads to a clinically significant reduction in glycated haemoglobin in people with existing diabetes [17], which is comparable to the effects of many commonly used oral hypoglycaemic agents [67]. Habitual consumption of diets with a low glycaemic index and/or load is associated with a significantly decreased risk of developing type 2 diabetes in high-quality observational studies [15,16].

In many parts of the world, total carbohydrate is a mandatory component of Nutrition Information/facts panels [68]. The total amount of available carbohydrate ingested as a single food accounts for 47–57% of the variability in blood glucose response (*p* < 0.05) [44]; however, in mixed meals, total carbohydrate is not significantly related to the mean capillary glucose (*p* = 0.10), plasma glucose (*p* = 0.20) or plasma insulin (*p* = 0.11) responses [69]. 

On the other hand, consideration of both the amount of total available carbohydrate and GI (i.e., glycaemic load) accounts for 85–94% of the variability in blood glucose in single foods [44] and ~90% of the variability in mixed meals [69]. Therefore, the total available carbohydrate content of a food alone is not as useful as GI and GL for people managing postprandial blood glucose levels.

The total sugars and total dietary fibre may also be considered to be markers of carbohydrate-quality and are, therefore, found on food Nutrition Information/Facts panels in many parts of the world. However, neither of these markers are useful tools for people with diabetes managing postprandial glycaemia [70]. The relationship between total sugars and blood glucose response is weak at best (r^2^ = 0.063), while there is no relationship between the total amount of fibre and postprandial blood glucose (r^2^ = 0.029) in either single foods or mixed meals [70].

Currently, where there is no food labelling with GI or GL, reducing total carbohydrate consumption is the only reliable method for reducing postprandial glycaemia and may explain some of the popularity of low-carbohydrate diets in recent years [71]. There is however generally little appetite for recommending low-carbohydrate diets among the dietetic profession owing to concerns that they can be nutritionally imbalanced, inconsistent with national dietary guidelines and without support from observational studies showing diabetes and other health risk reductions. 

While reducing excessive consumption of foods high in added sugars and refined starches is beneficial for people who over-consume them, reducing the ingestion of foods that are naturally high in minimally processed carbohydrates may not be beneficial and have unintended consequences. This is because minimally processed wholegrains, legumes, starchy vegetables and fruits are typically good sources of vitamins, minerals and dietary fibre and are associated with a lower risk of chronic diseases when compared with low carbohydrate diets [71]. While food composition can determine their nutrient content, only GI and GL can rank foods according to their ability to elevate blood glucose levels [7].

For the purposes of food labelling, the ICQC recommends GI labelling in preference to GL labelling. A food with a low GL may be one that is low in carbohydrates rather than having a low GI, when, in fact, the goal is to encourage the intake of high quality carbohydrate foods, particularly wholegrains and legumes. The nutrition guidelines of many countries now recommend wholegrains over refined grains [23,72], but many starch-rich wholegrain products are not low GI and may contribute to increased postprandial glycaemia. For people with type 2 diabetes, or at risk of developing type 2 diabetes and cardiovascular disease, reducing glycaemia without raising postprandial triglycerides, is an important day-to-day goal.

With the increased globalisation of the food supply, pre-packaged food is being sold in markets outside the original country of manufacture. While the Food and Agriculture Organisation of the United Nations does recommend minimal food labelling standards [68], these do not currently include provisions for GI. Foods manufactured in countries, such as Australia, New Zealand and Singapore that are making bona fide low GI claims (e.g., nutrition content or endorsements) may need to re-label them if they are to be sold in the European Union or Canada, to avoid breaking those nation’s food labelling laws. This inconsistent approach to labelling unnecessarily increases the cost of otherwise healthy foods for consumers, which, in itself, reduces the funding potentially available to improve the health and wellbeing among persons in impoverished communities.

Reducing the average dietary GI or GL is not complex: individuals do not need to do any calculations, they can simply swap low GI foods or beverages for regular high GI alternatives, within a food group/or category. When measured correctly, the GI method is precise enough to distinguish between high-GI and low-GI foods with 95% certainty [7]. 

In mixed meals, choosing a low GI option will lower the meal’s overall dietary GI, as low GI options always have a smaller effect on postprandial glycaemia when compared with higher GI alternatives, as the relative ranking does not change. For people with diabetes, most food classification systems group foods according to their macronutrient profile, so that carbohydrate exchanges can be made to facilitate consistent consumption of available carbohydrate throughout the day [73]. By choosing the lower GI options within a food category, individuals are also choosing the lower GL options.

Health messages to “swap it, don’t stop it” embedded within general healthy eating campaigns can encourage people to replace their current high-GI foods with healthier low GI alternatives to lower their average dietary GI and GL.

## 5. Conclusions

While there is consensus around the world that low GI foods, meals and diets are beneficial for people living with diabetes as well as those at risk, food regulation on GI labelling is inconsistent and contradictory. Successful implementation of Low GI labelling legislation permitting GI nutrition content claims and/or endorsements will require changes in regulation, public health education and increased adoption by the international food industry in order to have a meaningful impact on global health.

## Figures and Tables

**Table 1:** Food Standards Australia New Zealand’s Food Standards Code regulations for Glycaemic Index [2].

Glycaemic Index	(a) The Food Meets the NPSC [Nutrient Profiling Scoring Criterion] Unless the Food is a Food Standardised by Part 2.9 of the [Food Standards] Code; and (b) the Claim or the Nutrition Information Panel under Standard 1.2.8 Includes the Numerical Value of the Glycaemic Index of the Food.	
	**Low**	The numerical value of the glycaemic index of the food is 55 or below.
	**Medium**	The numerical value of the glycaemic index of the food is at least 56 and not exceeding 69.
	**High**	The numerical value of the glycaemic index of the food is 70 or above.

**Table 2:** South Africa’s Department of Health’s Glycaemic Index (GI) nutrition content claims.

GI Category Claim	Condition
Low GI	GI value: 0 to 55
Intermediate GI	GI value: 56 to 69
High GI	GI value: 70 and more

**Table 3:** Summary of low GI claims in Asia.

Countries	Use of Glycaemic Index Claims	Regulatory References
Cambodia	No regulation related to GI claim	
China	No regulation related to GI claim A “GI labeling specifications on pre-packaged foods” group standard is in progress. A Recommended industrial standard, WS/T 652-2019 *Standard for determination of food glycemic index*, was published in 2019 and is in force.	The Group standard development project (in progress) is led by the Chinese Nutrition Society The WS/T 652-2019 work was led by the National Health Commission of the People’s Republic of China.
India	Low GI claim permitted	Food Safety and Standards (Advertising and Claims) Regulation, 2018. India has its own standard for determination of food glycaemic index, Ref. IS 16495:2017
Indonesia	Test method protocol for GI determination was previously included in the 2011 claims regulation, but it has been removed in the latest 2016 claim regulation	BPOM Regulation NO HK.03.1.23.12.11.09909 (2011) regarding supervision of claims on processed food labels and advertisement. BPOM Regulation No.13 (2016).
Japan	No regulation related to GI claim	
Korea	No regulation related to GI claim	
Malaysia	No regulation related to GI claim	
Philippines	No regulation related to GI claim	
Singapore	Low GI claim permitted for several food categories under Healthier Choice Symbol scheme (cereals; legumes, nuts and seeds; convenience meals)	Singapore Guideline for Healthier Choice Symbol
Taiwan	No regulation related to GI claim	
Thailand	No regulation related to GI claim	
Vietnam	No regulation related to GI claim	

**Table 4:** Glycaemic index (GI) in European Dietary Reference Values (DRVs).

European Country	Dietary Reference Values on GI
France	The 2004 document from the French Agency ANSES concluded that the level of evidence is insufficient to provide indications on GI based on health benefits for the general population and prohibited the use of GI labelling or any derived measures [60]
Germany	The German Nutrition Society 2012 carbohydrate guideline document reported that: “*to date there is only possible evidence regarding a risk-increasing effect of high Glycaemic Index on some nutrition-related diseases. Therefore, no recommendations are made in that respect*” [61].
Nordic Countries	The Nordic Nutrition Recommendations 2012 concluded that “*There is not enough evidence that choosing foods with low Glycaemic Index will decrease the risk of chronic diseases in the population overall. However, there is suggestive evidence that ranking food based on their Glycaemic Index might be of use for overweight and obese individuals*” [62].
Italy	The 2014 DRVs from the Italian Society of Human Nutrition, included under “*Suggested Dietary Targets*” generic qualitative indications on preference for low-GI foods when intakes of carbohydrates approach the upper limit of intake, i.e., 60% energy. They also specified the need of preferentially selecting low GI foods provided the GI was not reduced by adding fructose and/or fat [63].
UK	In 2015, The Scientific Advisory Committee on Nutrition published a comprehensive opinion on carbohydrate and health [64]. Although it recognised that both lower GI and GL diets were associated with a decreased risk of type 2 diabetes, the Committee concluded that “*it is not possible to assign cause-effect relationships for outcomes based on variation in diet Glycaemic Index or Load, as higher or lower GI and GL diets differ in many ways other than just the carbohydrate fraction*”.

**Table 5:** Summary of GI claims and endorsements around the globe.

Country	GI Nutrition Content Claim in Food Regulations	GI Endorsement Program	Registered/Certified Trademark *	Comments
Australia	Yes, Since 2013. Nationally regulated.	Yes, since 2002.	Yes, Glycemic Index Foundation (Australia).	Low GI nutrient content claims, low GI Symbols are applied on a voluntary basis. Product must meet stringent nutrient criteria and the GI value must be measured in vivo by a GI testing laboratory according to either the Australian Standard (AS 4694—2007) or the International Standard (ISO 26642:2010).
Canada	No	No	Yes, Glycemic Index Foundation (Australia).	Diabetes Canada started work on endorsing a Low GI symbol in 2015.
China	Yes, since 2019. No national regulation.	In development.	In development, Chinese Nutrition Society.	
European Countries	No	No	Yes, Glycemic Index Foundation (Australia).	Reductions in postprandial glycaemia is considered a health claim. The health claim can only be incorporated into well-characterised food ingredients. A resolution motion on Low GI symbol program was sent to the EU Commission in 2018. Future potential to include GI in front-of-pack label in France. Currently, GI on food labels in Italy is not permitted in the absence of an authorised health claim for postprandial glycaemia for that food or one of its ingredients.
Hong Kong	No	No	Yes, Glycemic Index Foundation (Australia).	
India	Yes, Since 2018.	No	Yes, Glycemic Index Foundation (Australia).	
Indonesia	No	No	Yes, Glycemic Index Foundation (Australia).	
Japan	No	No	Yes, Glycemic Index Foundation (Australia).	
Malaysia	No	No	Yes, Glycemic Index Foundation (Australia).	
New Zealand	Yes, Since 2013. Nationally regulated.	Yes, since 2002.	Yes, Glycemic Index Foundation (Australia).	Low GI nutrient content claims, Low GI Symbols are applied on a voluntary basis. Product must meet stringent nutrient criteria, and the GI value must be measured in vivo by a GI testing laboratory according to either the Australian Standard (AS 4694—2007) or the International Standard (ISO 26642:2010).
Singapore	Yes	No	Yes, Glycemic Index Foundation (Australia).	Low GI claims are allowed in specific food categories with category-specific nutrient criteria.
South Africa	Yes, 2002–2011. Under review 2014—present.	Yes, Glycaemic Index Foundation of South Africa (GIFSA) since 2000.	GIFSA Trademark pending	A range of GI symbols (Very low GI; Low GI; Medium GI and High GI) are applied on a voluntary basis. Products must meet nutrient specification criteria, and the GI value must be measured according to the International Standard (ISO 26642:2010)
Taiwan	No	No	Yes, Glycemic Index Foundation (Australia).	
USA	No specific national regulations.	No	Yes, Glycemic Index Foundation (Australia).	GI is not defined by the United States Food and Drug Administration for regulatory labelling. As a result, GI claims may be permitted under the general false and misleading provisions of the Food, Drug and Cosmetic Act, which mandates that all labelling is truthful, evidence-based and not misleading.

* with low GI symbols.

## Data Availability

No new data were created or analyzed in this study. Data sharing is not applicable to this article.
